# Supplementary material for: A Global Screen for Assembly State Changes of the Mitotic Proteome by SEC-SWATH-MS
Source: Cell Syst. 2020 Feb 26;10(2):133–155.e6. doi: 10.1016/j.cels.2020.01.001 (PMC7042714; doi:10.1016/j.cels.2020.01.001)

P49736 | MCM2_HUMAN | MCM2 BM28 CCNL1 CDCL1 KIAA0030
Monomer MW [kDa]: 101.896 Monomer expected elution fraction: 40

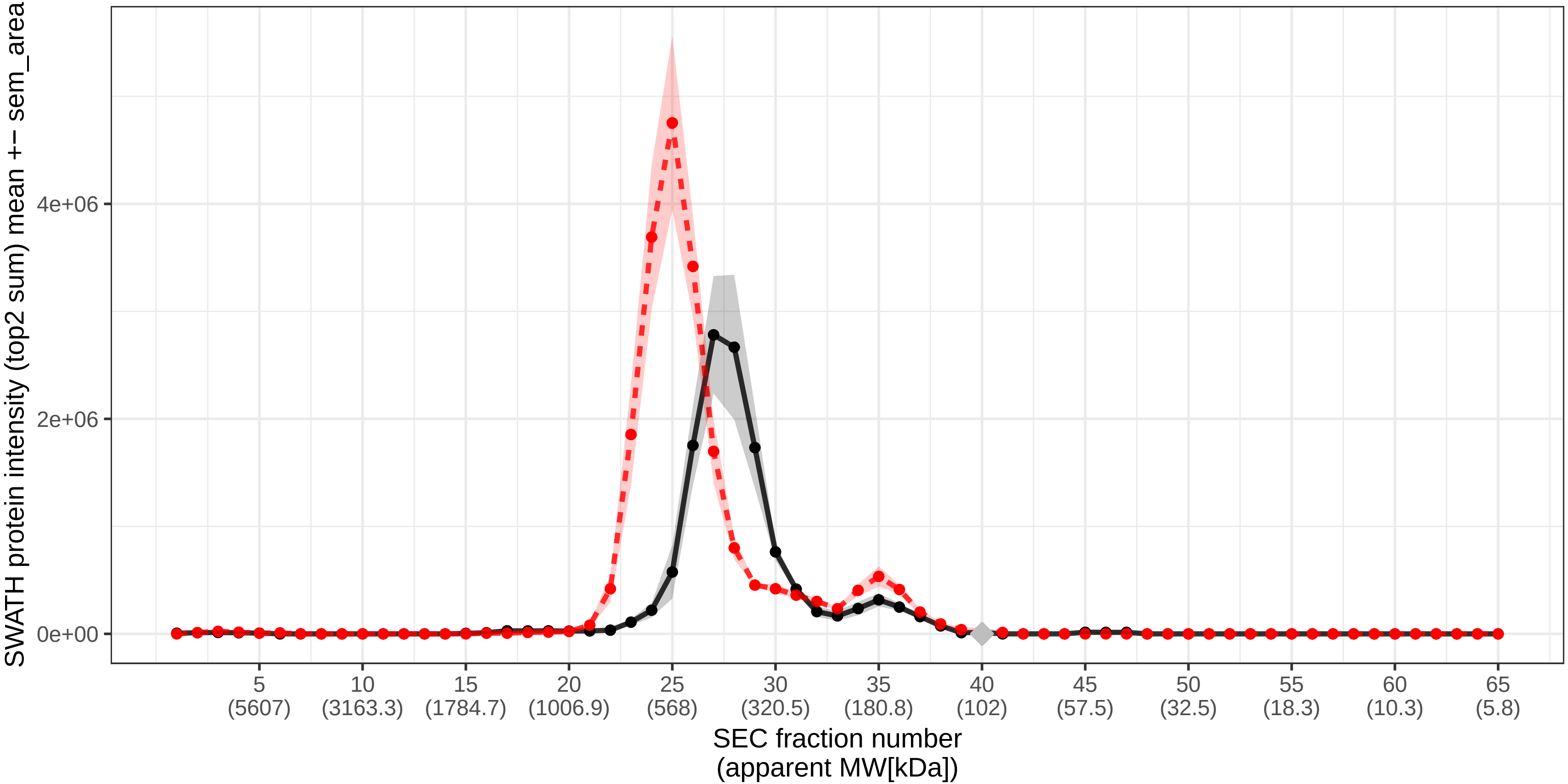

Supplement: Data S1. SEC-SWATH-MS Protein Chromatograms, Related to Figure 1 [file mmc6.zip › SECchrom_P49736_MCM2_HUMAN_MCM2_BM28_CCNL1_CDCL1_KIAA0030.pdf]
